# The effect of loading rate on the development of early damage in articular cartilage

**DOI:** 10.1007/s10237-016-0815-0

**Published:** 2016-08-11

**Authors:** J. M. Párraga Quiroga, W. Wilson, K. Ito, C. C. van Donkelaar

**Affiliations:** Orthopaedic Biomechanics, Department of Biomedical Engineering, Eindhoven University of Technology, P.O. Box 513, 5600 MB Eindhoven, The Netherlands

**Keywords:** Articular cartilage, Collagen damage, Loading rate, Finite element analysis

## Abstract

Experimental reports suggest that cartilage damage depends on strain magnitude. Additionally, because of its poro-viscoelastic nature, strain magnitude in cartilage can depend on strain rate. The present study explores whether cartilage damage may develop dependent on strain rate, even when the presented damage numerical model is strain-dependent but not strain-rate-dependent. So far no experiments have been distinguished whether rate-dependent cartilage damage occurs in the collagen or in the non-fibrillar network. Thus, this research presents a finite element analysis model where, among others, collagen and non-fibrillar matrix are incorporated as well as a strain-dependent damage mechanism for these components. Collagen and non-fibrillar matrix stiffness decrease when a given strain is reached until complete failure upon reaching a maximum strain. With such model, indentation experiments at increasing strain rates were simulated on cartilage plugs and damage development was monitored over time. Collagen damage increased with increasing strain rate from 21 to 42 %. In contrast, damage in the non-fibrillar matrix decreased with increasing strain rates from 72 to 34 %. Damage started to develop at a depth of approximately 20 % of the sample height, and this was more pronounced for the slow and modest loading rates. However, the most severe damage at the end of the compression step occurred at the surface for the plugs subjected to 120 mm/min strain rate. In conclusion, the present study confirms that the location and magnitude of damage in cartilage may be strongly dependent on strain rate, even when damage occurs solely through a strain-dependent damage mechanism.

## Introduction

Articular cartilage is a thin porous biphasic tissue that covers the ends of the bones in diarthrodial joints, allowing for a nearly frictionless relative motion. Its fluid phase accounts for 65–75 % of its wet weight. The solid phase consists of a fibrillar part and a non-fibrillar part, which are the viscoelastic collagen fibers and proteoglycans, respectively (Fithian et al. 1990). Among its main features, cartilage is responsible for carrying and distributing a great part of the loads developed in the joints, which can reach more than five times body weight in the knee (Bergmann et al. 2014). Cartilage successfully performs this load-bearing task partially by creating an internal fluid pressure and an osmotic swelling pressure due to the negatively charged proteoglycans (Fithian et al. 1990; Ateshian 2009; Párraga Quiroga et al. 2016). These internal pressures as well as the majority of the internal stresses are resisted by a strong collagen fibril network reinforcement that prevents the tissue from swelling and from undergoing large deformations. Hence, a healthy and functional collagen fibril network is a key factor for its mechanical behavior.

Despite its strong load-bearing properties, cartilage may become damaged when exposed to excessive loading. Self-repair is very limited and reduced cell viability, as a consequence of adverse loading most frequently observed in the superficial tangential zone (Chen et al. 2003), which will further reduce the repair capacity of cartilage. Due to this poor regenerative capacity, initial damage to the cartilage matrix often progresses into osteoarthritis when adverse loading conditions continue to prevail. Understanding cartilage damage progression is of paramount importance to early detection and to successfully treat early osteoarthritis, when tissue damage is still reversible. Even though both the fibrillar and the non-fibrillar matrix components are important to keep cartilage’s integrity at healthy levels, it is generally acknowledged that collagen damage is more important than proteoglycan loss, and it was shown that collagen damage is responsible for decreasing the tensile stiffness of osteoarthritic cartilage (Bank et al. 2000). It has been proposed that collagen fiber strains are good predictors of cartilage collagen damage (Wilson et al. 2007). Collagen damage may initiate at the surface (Hollander et al. 1995), although there is also experimental evidence with numerical support that it can start in a sub-superficial region and penetrate to the surface at a later stage (Wilson et al. 2006a, b; Hosseini et al. 2014). Differences in appearance of cartilage damage may result from differences in applied loading rates. On the one hand, more cell death occurs at lower loading rates, which can be explained due to the fact that there is more time for relaxation so fluid is expelled and more deformation is reached, thus compressing the cells to larger magnitudes. On the other hand, release of proteoglycan into the culture media and the depth of surface lesions increased with higher loading rates (Ewers et al. 2001). It can be speculated that collagen damage occurs prior to non-fibrillar matrix damage; consequently, the proteoglycans can escape from the collagen network where they were constrained (proteoglycan depletion). Due to proteoglycan depletion, less osmotic pressure will be generated and therefore more compression can be expected, which can lead to proteoglycan damage.

The above results suggest that damage mechanisms in cartilage constituents may be loading-rate-dependent. However, the biphasic nature of cartilage and the viscoelastic properties of the collagen fibrils together result in time-dependent distributions of strains in the tissue. In addition, the non-homogenous distribution of the cartilage constitutive components and the varying anisotropy throughout its thickness make the internal mechanical conditions complex to understand (Wang et al. 2002; Chen et al. 2001). Consequently, the distributions of strains and stresses, the locations of excessive peak values and the changes in these parameters over time are difficult to predict.

The present study postulates that the time-varying distribution of peak strains in the tissue may result in different damage responses in cartilage that is loaded at different loading rates. Consequently, it predicts loading-rate-dependent differences in damage profiles, even with a damage mechanism that is only strain-dependent, but not strain-rate-dependent. The present study questions whether the above premise holds and aims to illustrate the theoretical effect on location and amount of damage to both the collagen network and the non-fibrillar matrix.

For this purpose, the study employs a former cartilage damage model (Hosseini et al. 2014). This model contained a local damage mechanism, which may theoretically result in mesh-dependent localization of damage. A secondary aim of the present study is to update the latter damage model with a non-local description of damage mechanics.

## Methods

### Cartilage model

A swelling, collagen fibril reinforced, poro-viscoelastic cartilage FE model (Wilson et al. 2006a) implemented through a UMAT subroutine in Abaqus (Abaqus 6.13, Dassault Systèmes, 2013) was used. In this model, the total cartilage stress at each integration point is given by Eq. 1:

1 $$\begin{aligned} \sigma _{tot}= & {} -\mu _f \mathbf{I}+\frac{n_{s,0} }{J}\left( \left( 1-\sum _{i=1}^{totf} {\rho _c ^{i}}\right) \sigma _{nf} +\sum _{i=1}^{totf} {\rho _c ^{i}\sigma _{f } ^{i}} \right) \nonumber \\&-\Delta \pi \mathbf{I} \end{aligned}$$

where $\mu _{f}$ is the fluid pressure, **I** is the unit tensor, *J* is the volumetric deformation tensor which equals the determinant of the deformation gradient tensor **F**, totf is the number of fibril orientations considered at each location, $\Delta \pi$ is the osmotic swelling pressure, $\sigma _{f}$ is the stress in the collagen fiber network, $\sigma _{nf}$ is the stress in the non-fibrillar network (proteoglycans), $\uprho _{\mathrm{c}}$ is the volume fraction of the collagen fibers which depends on the direction of fibril *i* with respect to the total volume of the solid matrix, and $n_{s,0}$ is the initial solid volume fraction. The non-fibrillar network models a 3D isotropic continuum using a modified neo-Hookean model (Eq. 2). For more details and a derivation of this equation, see (Wilson et al. 2007). The stress in the non-fibrillar network is given by Eq. 2, where $Gm_{nf}$ is the shear modulus of the non-fibrillar matrix.

2 $$\begin{aligned} \sigma _{nf}= & {} -\frac{1}{6}\frac{\ln (J)}{J}Gm_{nf} \mathbf{I}\left[ -1+\frac{3(J+n_{s,0} )}{(-J+n_{s,0} )}\right. \nonumber \\&\left. +\frac{3\ln (J)Jn_{s,0} }{(-J+n_{s,0} )^{2}}\right] +\frac{Gm_{nf} }{J}(\mathbf{F}\cdot \mathbf{F}^\mathrm{T}-J^{2/3}{} \mathbf{I}) \end{aligned}$$

The stress in the collagen network is given by:

3 $$\begin{aligned} \sigma _f =\sigma _{fib} \vec {e}_f \vec {e}_f +\sigma _{f \,iso} \end{aligned}$$

**Fig. 1:**
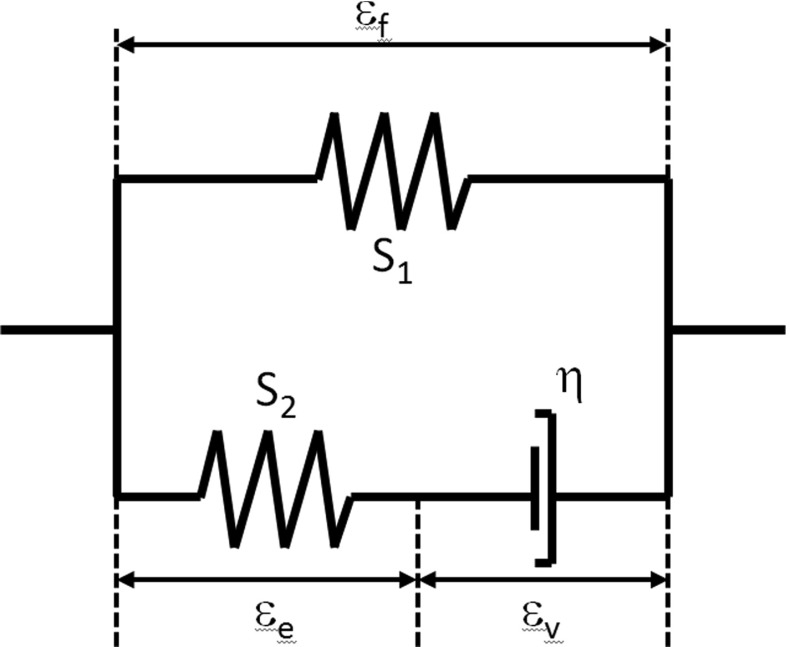
Standard linear solid model representing collagen fiber behavior; with $\varepsilon _{\mathrm{f}}$ the total fibril strain, $\varepsilon _{\mathrm{v}}$ the dashpot strain and $\varepsilon _{\mathrm{e}}$ the strain in spring $S_{2}$, where $S_{1}$ and $S_{2}$ are the stiffness of the springs and $\eta$ is the viscoelastic constant of the dashpot

**Fig. 2:**
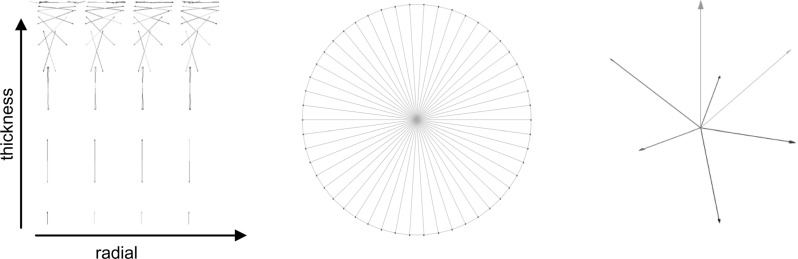
*Left* direction of primary collagen fibers, running in *vertical* (thickness) direction and bending to be parallel to the AC surface (*radial* direction) when reaching the top layers. *Center* Top view of a cartilage plug showing split line pattern of primary fibers oriented in *radial* direction. *Right* secondary collagen fibers at each integration point; seven directions were considered running at $45^{\circ }$ of each other

where $\sigma _{fib}$ is described by Eqs. 4–6 in combination with Fig. 1. $\sigma _{f \; iso }$ represents the isotropic stiffness of the fibers and is described with the same neo-Hookean model used to describe the stress in the non-fibrillar network ($\sigma _{nf}$, Eq. 2). The only difference is that $Gm_{nf}$ *is replaced by Gmf which is* the shear modulus of the collagen fibers and was obtained from the literature $Gm_{f}=0.01144$, (Barthelemy et al. 2015). The values for $E_{1}, S_{1}, E_{2}, S_{2}, \eta , Gm_{nf}$ and $Gm_{f}$ were previously obtained from fitting the numerical model to experimental data (Párraga Quiroga et al. 2016).

4 $$\begin{aligned} \sigma _{fib} =\frac{\lambda }{J}P_f \vec {e}_f \vec {e}_f \end{aligned}$$

with $\lambda$ representing the elongation of the fibril and $\vec {e}_f$ the unit vector in the current fibril direction. The total fibril stress, characterized by the nonlinear viscoelastic solid model shown in Fig. 1, is calculated as:

5 $$\begin{aligned}&P_f =P_1 +P_2 \end{aligned}$$

6 $$\begin{aligned}&\begin{array}{ll} P_1 =E_1 (e^{S_1 \varepsilon _f }-1) &{}\hbox {for}\quad \varepsilon _f>0 \\ P_1 =0 &{}\hbox {for}\quad \varepsilon _f \le 0 \\ P_2 =E_2 (e^{S_2 \varepsilon _e }-1)=\eta \dot{\varepsilon }_v &{}\hbox {for}\quad \varepsilon _e >0 \\ P_2 =0&{}\hbox {for}\quad \varepsilon _e \le 0 \\ \end{array} \end{aligned}$$

The fibril network is defined by viscoelastic fibers oriented in 9 different directions: 2 primary, dominant directions describing the arcade-like structure (Benninghoff 1925) and seven secondary orientations (with lower fiber density). The orientation of both families of fibers is shown in Fig. 2. In the latter figure, three regions can be distinguished:

- deep zone (0.0–0.7 normalized height), here the fibers are oriented perpendicular to the bottom surface;
- middle zone (0.7–0.975 normalized height), in this region the fibers changed it orientation from perpendicular to the bottom to parallel to the surface; and
- superficial zone (0.975–1 normalized height) where the fibers are oriented parallel to the surface.

The osmotic swelling pressure is calculated according to Eq. 7,

7 $$\begin{aligned} \Delta \pi =\phi _{int} RT\sqrt{c_{F,exf}^2 +4\frac{\gamma _{ext}^{\pm {2}}}{\gamma _{int}^{\pm {2}}}c_{ext}^2 }-2\phi _{ext} RTc_{ext} \end{aligned}$$

where $\phi _{int} /\phi _{ext}$ are the osmotic coefficient, *R* is the gas constant (8.3145 J/(mol K)), *T* is the absolute temperature (293 K), $c_{F,exf}$ is the effective fixed charge density, $\gamma _{ext}^\pm /\gamma _{int}^\pm$ are the activity coefficients, $c_{ext}$ is the external salt concentration, the reader is referred to Huyghe et al. (2003) for more details about these coefficients.

The permeability of the tissue is calculated according to Eq. 8,

8 $$\begin{aligned} k=\alpha (1-n_{exf} )^{-M} \end{aligned}$$

where *M* and $\alpha$ are material constants, $n_{exf}$ is volume fraction of the extra fibrillar water, see Wilson et al. (2006a, (2006b) a for more details.

The fluid fraction $\hbox {n}_{\mathrm{f},0 }(\hbox {n}_{\mathrm{f},0 }+ \hbox {n}_{\mathrm{s},0} =1)$, the collagen fraction $\rho _{\mathrm{c}}$ and the fixed charged density $C_{{F,exf}}$ were obtained from Wilson et al. (2006a, (2006b) and are described below as a function of the normalized depth(z):

9 $$\begin{aligned}&n_f =-0.2z+0.9 \nonumber \\&\rho _{c,tot} =1.4z^{2}-1.1z+0.59 \nonumber \\&C_{F,exf} =-0.1z^{2}+0.24z+0.035 \end{aligned}$$

**Fig. 3:**
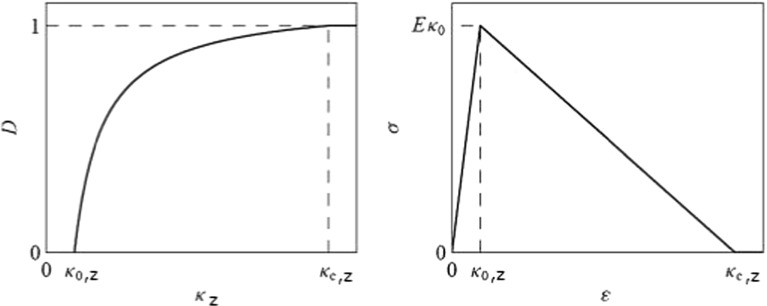
Evolution of damage (*D*) and schematic of stress ($\sigma )$ showing linear softening in the constituent as a function of strain history variable ($\kappa _{z})$ from the moment of activation ($\kappa _{0,\mathrm{z}})$ until complete failure ($\kappa _{\mathrm{c,z},} \textit{D}\,=\,1$)

### Non-local versus local damage theory

An algorithm for cartilage damage development was previously implemented in the aforementioned FE model (Hosseini et al. 2014). This local damage theory could result in damage localization during which all damage concentrates in a vanishing volume. According to well-established damage mechanics theories (Pijaudier-Cabot and Bažant 1987; de Vree et al. 1995), this could cause the material to behave in an extreme brittle fashion, failing at almost zero energy (Peerlings et al. 2002). To prevent such localization, a non-local theory (Pijaudier-Cabot and Bažant 1987; Pijaudier-Cabot et al. 1988; Borst and Mühlhaus 1992; Peerlings et al. 2002), was implemented in the present cartilage damage model. Differences between the local and the non-local damage model were investigated and will be shown in the results section.

In contrast to the local method in the non-local method not the local strain in each integration point is used, but a strain averaged over a certain volume around the integration point. For the averaging, a Gauss weighting function is used (Eq. 10). This means that the closer a point lies to the integration point of interest, the larger its contribution to the averaged strain. To obtain a mesh-independent solution, enough integration points should lie within the averaging volume, which is specified by the characteristic length.

10 $$\begin{aligned} \psi _0 (\vec {x},\vec {y})=\frac{1}{(2\pi )^{3/2}l^{3}}{\varvec{exp}}\left[ {-\frac{\left\| {\vec {x}-\vec {y}} \right\| }{2l^{2}}} \right] \end{aligned}$$

where *l* is a length parameter, referred to as the characteristic length, which is a material property related to the scale of the microstructure. In this way, the influence of a result variable depends on the distance to the location of interest. Results may be mesh-dependent if few integration points are within the characteristic length, but becomes mesh-independent if enough integration points are present within the characteristic length.

The weighing function then multiples the local strain history variable in each collagen fiber to account for the strains of those fibers oriented in the same direction (fiber 1$\rightarrow$9), which are included within a given characteristic volume (determined with the characteristic length). In this way, the strains are not localized but smeared out over a given volume and therefore reduced.

In this study, the characteristic length was chosen to be 0.025 mm so that it matched the thickness of the superficial tangential zone beyond which the fiber orientation changes considerably. The mesh was refined until the element size was smaller than the characteristic length and the resulting damage progression was independent of further mesh refinement.

In the current constitutive model, no strain-rate dependencies have been included and only strain-dependent damage is implemented. Thus, damage (*D*) in the solid part of the tissue starts as soon as the level of collagen fibril strain exceeds a threshold. It grows from zero to one, where the fibrils reach their rupture point ($\varepsilon = 0.22$). Damage factor *D* follows a linear damage evolution law (Eq. 11).

11 $$\begin{aligned} D=\left\{ {{\begin{array}{l} 0 \\ {\frac{k_{C,Z} }{k_Z }\frac{k_Z -k_{0,Z} }{k_{C,Z} -k_{0,Z} }} \\ 1 \\ \end{array} }{\begin{array}{ll} &{}\hbox {if}\quad k_Z \le k_{0,Z} \\ &{}\hbox {if}\quad k_{0,Z}<k_Z <k_{C,Z} \\ &{}\hbox {if}\quad k_Z \le k_{C,Z} \\ \end{array} }} \right. \end{aligned}$$

where $k_{Z }$ is the strain history variable which corresponds to the strain in the collagen fibrils and the deviatoric strain for the non-fibrillar matrix (Eq. 12); $k_{0,\mathrm{Z}}$ is the strain at which fibril damage initiates ($k_{0,\mathrm{Z} }= 0.06$); $k_{\mathrm{C,Z}}$ is the value for complete failure of the fibril ($k_{\mathrm{C,Z}} = 0.22$). The values for damage initiation and complete failure in the other solid component: non-fibril matrix (proteoglycan), are 0.3 (initiation) and 0.6 (failure). The threshold for damage initiation and complete failure were adapted from a previous study (Hosseini et al. 2014) and updated to accommodate for the averaging effect over the characteristic length (Eq. 10).

12 $$\begin{aligned} \varepsilon =\frac{1}{3}\sqrt{(\varepsilon _1 -\varepsilon _2 )^{2}+(\varepsilon _1 -\varepsilon _3 )^{2}+(\varepsilon _2 -\varepsilon _3 )^{2}} \end{aligned}$$

where $\varepsilon _{1}, \varepsilon _{2}$ and $\varepsilon _{3}$ are the principal strains.

With an increase in the fraction of degraded collagen or non-fibrillar matrix, represented by an increase in *D*, the homogenized tissue stress $\sigma$ decreases (Fig. 3) according to Eq. 13 and the applied external loading is redistributed to the rest of the tissue.

13 $$\begin{aligned} \sigma =(1-D){*}\tilde{\sigma } \end{aligned}$$

where $\tilde{\varvec{\sigma }}$ is the effective tissue stress.

Finally, the damage parameter for each fiber ($D_{1\rightarrow 9})$ obtained from Eq. 11 is weight averaged for the 9 fibrils (2 primary and 7 secondary), according to Eq. 14, where *C* = 3 is the relative density of the primary to secondary fibers (Wilson et al. 2006a).

14 $$\begin{aligned} \textit{D}=\frac{\textit{C}(\textit{D}_1 +\textit{D}_2 )+\textit{D}_3 +\textit{D}_4 +\textit{D}_5 +\textit{D}_6 {+}{} \textit{D}_7 {+}{} \textit{D}_8 {+}{} \textit{D}_9 }{2\textit{C}\,+\,7}\nonumber \\ \end{aligned}$$

A summary of all the previously parameters that are necessary input to the constitutive model is given in Table 1.

**Table 1:** Summary of necessary input to the model

Material parameter	Description	Value	Reference
$Gm_{nf}$	Shear modulus of the PG matrix (MPa)	0.7722	Párraga Quiroga et al. (2016)
$Gm_{f}$	Shear modulus of the collagen matrix (MPa)	0.01144	
$\hbox {E}_{1}$	Material constant (elastic fibrilpart) (MPa)	4.362	
$S_{1}$	Material constant (elastic fibrilpart) (−)	14.39	
$\hbox {E}_{2}$	Material constant (viscoelastic fibrilpart) (MPa)	20.25	
$S_{2}$	Material constant (viscoelastic fibrilpart) (−)	43.96	
$\eta$	Dashpot of viscoelastic fibrilpart (MPa s)	153,200	
$\alpha$	Permeability constant ($\hbox {mm}^\wedge$ 4/(N s))	1.767e−4	Wilson et al. (2006a)
*M*	Nonlinearity term of permeability (−)	1.339	
$k_{0,\mathrm{z}}$ (fibril)	Fiber damage initiation strain	0.06	Adapted from Hosseini et al. (2014)
$k_{\mathrm{c, z} }$(fibril)	Fiber damage failure strain	0.22	
$k_{0,\mathrm{z}}$ (non-fibril)	Matrix damage initiation strain	0.3	
$k_{\mathrm{c, z}}$ (non-fibril)	Matrix damage failure strain	0.6	
*C*	Ratio of primary to secondary fibers	3	Wilson et al. (2006a)

### FEA simulations

Damage progression in both the collagen and the non-fibrillar matrix was monitored in cartilage plugs of 5.5 mm diameter and 1 mm thickness that were loaded with a 1-mm-radius rigid hemispherical-tip indenter at different loading rates (5, 15, 60 and 120 mm/min) until 30 % axial compression was reached. The cartilage plug was constrained in all directions in the bottom. Fluid was allowed to flow in all free surfaces (side and top surface). In the top surface below the indenter the area where free fluid flow was allowed changed during the progression of the analysis as the indenter travelled downward. In order to control this complex boundary condition, the Abaqus user subroutines are FRIC and FLOW. As shown in Fig. 3 as soon as damage starts (Fig. 3 left), the material softens, which is depicted by the reduction in the stress (Fig. 3 right). The onset of damage might start at some moment while the indenter is traveling downward, and due to the damage progression characteristics, the behavior of the tissue will change (soften) as more damage is detected. First, the implementation of the non-local damage theory was tested by evaluating damage development in meshes of various mesh densities. A mesh density for further simulations was chosen such that both damage evolution and the distribution of mechanical parameters were mesh-independent. Further simulations used the selected mesh density. Simulations were performed on a quarter of the sample to reduce computation times. Axisymmetric modeling was not viable because of limitations to implement the Gauss weighing function (Eq. 7) in the direction of revolution, i.e., it would have been possible to average results in plane, but out of plane there would have been no results to be considered for averaging. Second, the progression of damage in the collagen fibers and in the non-fibrillar, proteoglycan-rich matrix was monitored over time while loading was applied at the four different loading rates.

**Fig. 4:**
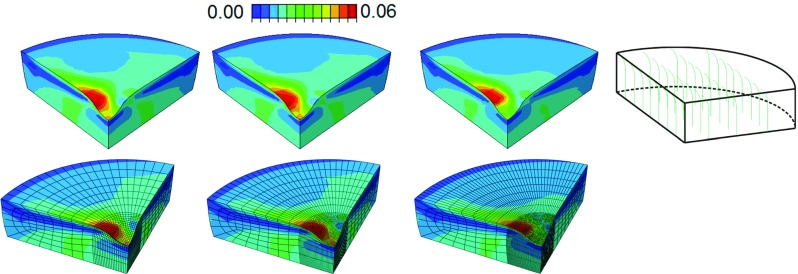
Mesh sensitivity analysis. One quarter of cartilage collagen plug under unconfined indentation compression. Variable depicted is collagen fibril strain in one primary fiber direction (see schematic orientation in the *left*). Three meshes were evaluated: 4855, 9812, 19431 C3D8RP elements were used in each case, *top row* from *left* to *right*. On the *top row*, the mesh is turned off for better strain visualization. The *bottom row* shows the mesh refinement in the indented area

**Fig. 5:**
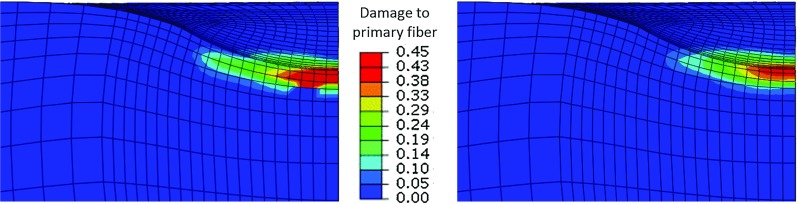
Damage in one of the primary collagen fibers predicted with a local damage approach (*left*) and with a non-local damage approach (*right*). 20 % compression was prescribed

### Mesh density

Simulations with several mesh densities and the same characteristic length showed no significant differences in profiles of damage development (Fig. 4). A mesh with intermediate density was chosen (Fig. 4b; 9811 C3D8P elements) for further simulations of damage evolution. Independent of the fiber analyzed, either a single fibril or the average of all 9 fibers (Eq. 10), the results were independent of the mesh; only results for collagen strain in the first fibril are shown.

## Results

Damage in one of the primary fibers was evaluated at 20 % compression both with the local (Hosseini et al. 2014) and the currently introduced non-local-damage approach, as shown in Fig. 5. These results clearly show how the local damage gives larger damage areas and localizes to the elements, whereas the non-local approach shows lower values as a results of the weight-averaging damage based on Gauss function (Eq. 7).

**Fig. 6:**
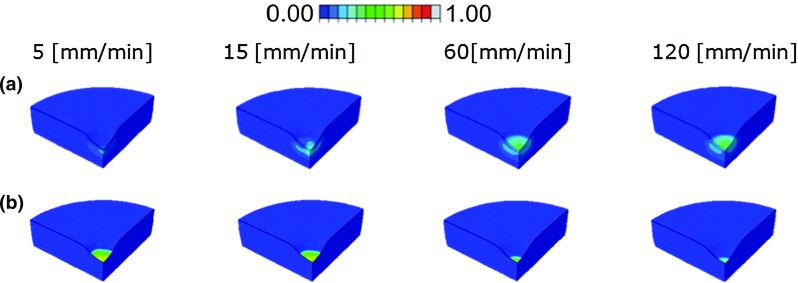
Damage in the collagen fibers (**a**) and damage in the matrix (proteoglycan) (**b**) when compressing the tissue to $\sim 30\,\%$ of its height. Loading rate increases from left to right: 5, 15, 60 and 120 mm/min

**Fig. 7:**
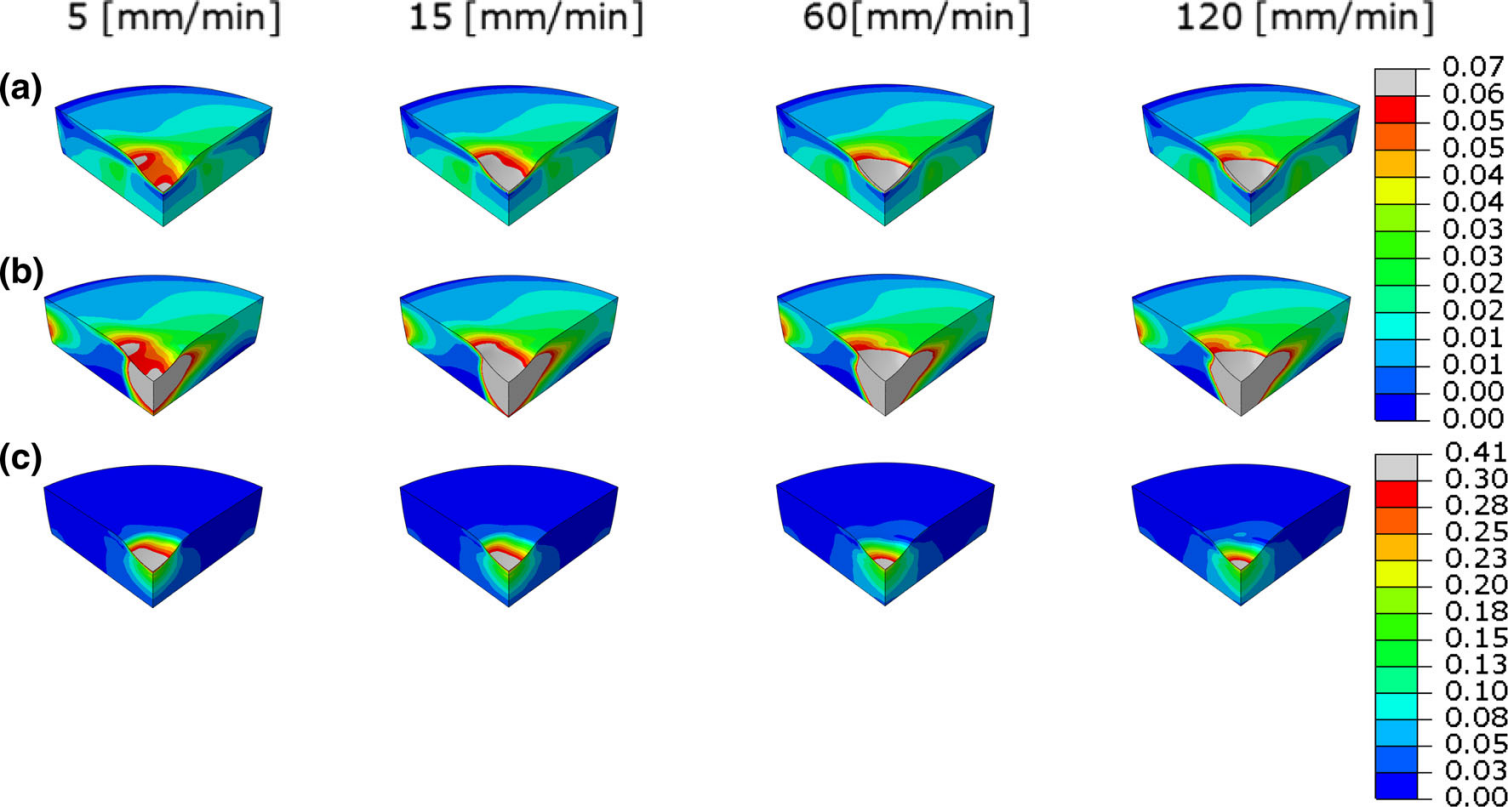
**a**, **b** strains in two out of nine collagen fibril, fibril 1 and 3, respectively. Limit in the legend has been set to a maximum of 0.06 ($k_{0,\mathrm{z}}$ fibril) to show the areas where damage has started. **c** strain in the non-fibrillar matrix, similarly with the strains set to a maximum of 0.3 ($k_{0,\mathrm{z}}$ non-fibril)

The volume of collagen fiber network damage increased with strain rate until 60 mm/min, when it reached its maximal size (green volume in Fig. 6a). The maximum strain in the collagen fibers increased until this same strain rate (60 mm/min) after which a plateau seemed to have been reached. The latter effect was addressed in a previous study (Párraga Quiroga et al. 2016). Also, the maximum amount of damage increased from 21 % at 5 mm/min to 42 % at 120 mm/min strain rate (Fig. 6a). In contrast, the damage volume of the non-fibrillar proteoglycan-rich matrix was largest at the lowest loading rate (5 mm/min), with 72 % of the matrix being damaged under the indenter tip and decreasing to only half that amount (34 %) for the fastest, 120 mm/min, loading rate (Fig. 6b). Damage in the fibrils is averaged for the nine fibers according to Eq. 14; nonetheless, Fig. 7 shows the strains in one principal fiber and one secondary fiber to give more insight on the level of strains and those regions that according to the strain values of damage initiation $k_{0,\mathrm{z}}$ will start to develop damage. Also the same is represented for the non-fibrillar matrix.

**Fig. 8:**
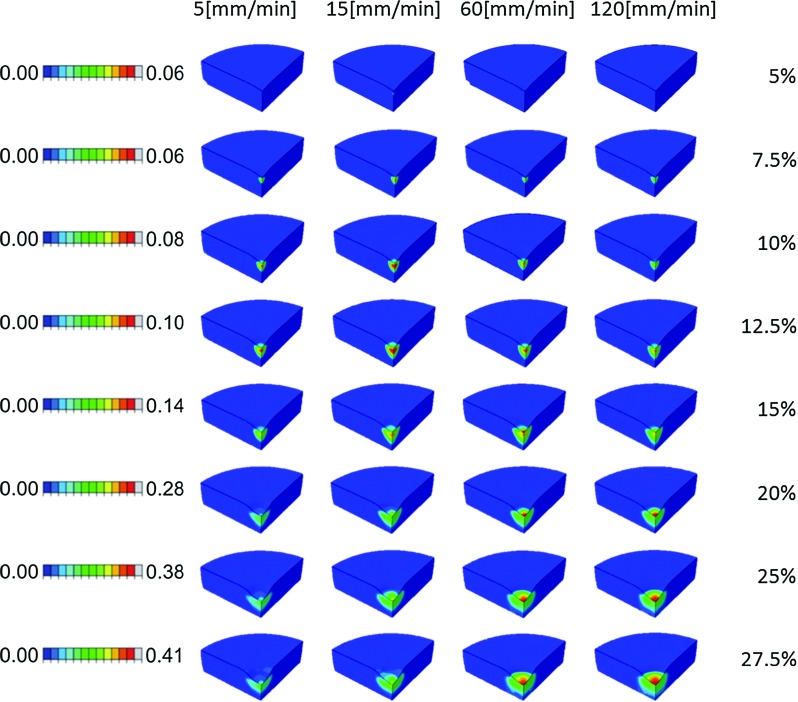
Progression of collagen fiber damage when the compression magnitude increases from 5 [%] to 27.5 [%]. The strain rate increases from left to right: 5, 15, 60 and 120 mm/min. Note that the maximum limit on the legend is updated in each row of images

Damage progression in the non-fibrillar matrix increased steadily in both magnitude and size with increasing indentation depth, starting at the indenter tip. Damage progression in the collagen matrix followed a more interesting profile, which varied in location depending on the applied strain rate (Fig. 8). The damage started to develop in a region away from the surface, at a depth of approximately 20 % of the sample’s height. This location coincided with the middle zone of the cartilage, where the fibers change orientation from perpendicular in the deep zone to parallel with the articular surface in the superficial zone. This early, deep damage was more pronounced for the lower and intermediate loading rates between 10 and 15 % compression, reaching approximately 10 % local tissue damage. Above 15 % strain, damage became more pronounced at the surface, where eventually 40 % of all collagen under the indenter was damaged (Fig. 8). In general, it could be concluded that at higher strain rates, most of the damage seemed to be confined to the surface and upper regions of the cartilage.

## Discussion

Using a non-local damage theory, this study explored damage development in cartilage when ramp loading was applied. The hypothesis was that loading-rate-dependent cartilage damage would occur because of the time-dependent behavior of cartilage, even when the damage mechanism was not strain-rate-dependent. This hypothesis was confirmed for both the non-fibrillar proteoglycan-rich matrix and the collagen network. Interestingly, collagen damage increased with strain rate, whereas non-fibrillar matrix damage decreased at higher loading rates. The rate dependency for collagen showed most peculiar effects; it is more pronounced in the middle zone for smaller loading magnitudes and lower loading rates, and in the superficial zone for faster loading rates (Fig. 8). In conclusion, both location and magnitude of damage are loading-rate-dependent, and this dependency is different for proteoglycan-rich matrix and the collagen network.

The damage development that is predicted in the present study concurs with experimental data:

- It has been shown that superficial collagen damage occurs at higher loading rates. Macroscopic lesions in cartilage are more severe when the loading velocity increases (Ewers et al. 2001), and superficial cracks only occurred at loading rates above $0.7\,\times \,10^{-3} s^{-1}$ during unconfined compression (Morel and Quinn 2004). Each of the loading rates in the present study exceeded this critical level (5–120 mm/min, equivalent to 0.083–2.0 $s^{-1}$ for 1 mm thick cartilage). However, the nature of the loading (indentation vs. unconfined compression) makes it difficult to make direct comparisons. During indentation, the local compressive, tensile and shear strains may be higher under the indenter than in an unconfined compression experiment, whereas to reach similar displacements larger forces are required in unconfined compression (Párraga Quiroga et al. 2016).
- The computed pattern of collagen damage development concurs with the literature. In agreement with Rolauffs (Rolauffs et al. 2010) damage localizes in the top quarter of the tissue and does not extend into the deep zone. Collagen damage starts in the sub-superficial region and progresses toward the surface upon more excessive loading. This peculiar pattern was already suggested by Hollander (Hollander et al. 1995), who found more damage in the mid-to-deep zones compared to the surface and was later confirmed by Wilson (Wilson et al. 2006b), who correlated the severity of collagen damage with cartilage thickness.
- The pattern that non-fibrillar matrix damage starts at the surface and progresses slowly into the sub-superficial deeper areas correlates very well with experimental data (Lin et al. 2004).
- The present paper predicts that damage to the non-fibrillar matrix is more apparent for lower than for higher strain rates (Fig. 6b). Unfortunately, this finding cannot be correlated directly with experimental data. However, assuming that chondrocytes may not survive the mechanical conditions that induce non-fibrillar matrix damage, cell viability may serve as an indirect measure for areas of excessive non-fibrillar matrix strain. Indeed, in agreement with the model predictions, cell viability decreases with reduced compression rates (Ewers et al. 2001), and cell death has been found in tissue that was subjected to low compression rates, but not for similar loads applied at higher rates (Morel and Quinn 2004). Obviously, it remains speculative whether cell viability can be used as a marker for the location of matrix damage. Yet, it is possible that proteoglycan damage and cell death are triggered by the same mechanical stimuli, or that cell death follows matrix damage.
- Sadeghi et al. (2015) Investigated the effect of frequency on superficial cartilage cracks. In their study, they showed cracks on the surface increasing its length as the loading frequency increased (1–10–100 Hz). Their repetitive load applied at 1 Hz (somehow comparable with our 15 mm/min loading) did not show damage, comparable with our results for 15 mm/min where little damage is observed. At 10 Hz (comparable with 120 mm/min), they observed cracks increasing its length up to 3 mm. In our case, almost 30 % damaged is observed for comparable conditions.
- Last, the optimum would be to compare the thresholds used here with other experimental/numerical values. In this regard, other authors have also presented a threshold to determine damage onset: Mononen et al. (2016) used a value of 7 MPa for the maximum principal stresses. With a very similar description of the constitutive model, yet the model is different because different time dependencies and different loading rates have been used. Thus, comparing such thresholds would not be fair. Furthermore, no osmotic swelling has been included in such model, and it has been recently shown how important this component is for cartilage mechanics (Párraga Quiroga et al. (2016)).

Five effects require further explanation. First, the amount of damage increased with increasing loading rate. This is explained by the loading-rate-dependent collagen fiber strains that occur in cartilage (Párraga Quiroga et al. 2016; Hosseini et al. 2014). Lower loading rates will allow for fluid flow and relaxation of the viscoelastic collagen fibers. Therefore, stresses and strains in the collagen are less localized, which results in increased volumes with elevated strains, yet with lower maximum collagen strains. If these strains remain below the damage threshold, damage will not occur. Under higher loading rates, the strains cannot distribute and locally remain higher. If they exceed the threshold, damage initiates. The resulting softening allows further increase in the local strain, which may cause further damage. This explains why collagen damage is most severe at the surface for higher loading rates.

Second, the initiation of damage in the deeper zones is explained from the tangential orientation of the dominant collagen fibers in the superficial zone. Under indentation loading, the surface is strained in the direction parallel to the surface. The collagen effectively counters this deformation at the surface. Therefore, the strains remain below the threshold during the early phase of loading, and surface collagen does not damage. Below the surface, however, the fraction of tangential collagen fibrils decreases. Consequently, the stress in the tangential direction will strain the tissue more. The strain threshold is exceeded and damage initiates in the transitional zone, i.e., below the cartilage surface.

Third, this sub-superficial damage in the transitional zone is more excessive for the lower loading rates. This is again explained by the viscoelastic properties of the collagen. At lower loading rates, the collagen in the surface is given slightly more time to strain, and therefore, the sub-superficial area is allowed to deform slightly more. Thus, at lower loading rates the collagen in the transitional zone will exceed the damage threshold earlier than it would at higher loading rates.

Fourth, damage in the non-fibrillar matrix occurs at the surface and not in the same region where collagen damage is found. Collagen damage develops in areas with large tensile strains in the fiber direction, whereas matrix damage is hypothesized to result from excessive deviatoric strains. The latter strains are highest in the surface, because this volume is elongated in the tangential direction and maximally compressed in the perpendicular direction. Thus, the ratio of compression to elongation is largest in the superficial tangential zone.

Fifth, in contrast to collagen damage, damage in the non-fibrillar matrix increases with lower loading rates. Deviatoric strains increase when both the tangential elongation and the perpendicular compression increase. This is the case when the tissue is given more time to relax, i.e., at lower loading rates. Therefore, matrix damage is more pronounced with lower loading rates.

A non-local damage model is implemented and employed for the first time to study damage in cartilage tissue. There are some limitations to the interpretation. First, the threshold values for damage initiation were taken from a former local damage model (Hosseini et al. 2014), where they were derived from experimental data. However, that study discussed that relevant data exist neither for collagen type II nor for proteoglycan-rich matrix in cartilage. The threshold values were transferred to the present implementation by accounting for the averaging procedure over the characteristic length (Eq. 10). Better validation of the threshold values will be performed when experimental data become available. Second, the characteristic length was chosen equal to the thickness of the superficial tangential layer, the smallest typical length scale for cartilage matrix. Although this is a reasonable choice, this volume does not allow detection of damage gradients within the superficial zone, if such gradients would occur. To do so, it would be possible by decreasing the characteristic length and using a finer mesh, or by using ellipsoidal volumes with higher aspect ratio in the direction of the collagen fibrils.

The simulations are indentations with a small indenter on a circular, osteochondral explant. These conditions do not represent physiological in vivo cartilage loading. However, it has several advantages. First, indentation loading reduces adverse and uncontrollable effects of the boundaries of the explant, which are damaged as a consequence of drilling and sample preparation. Second, indentation induces more local elongation in the tissue than unconfined compression, and these positive strains are considered the cause of fiber damage. Third, indentation produces strain gradients in vertical and horizontal directions, whereas horizontal strains during unconfined compression are uniform within each zone. Therefore, indentation provides more information on damage progression patterns, providing more possibilities for future validation.

In conclusion, using a non-local damage model, this study demonstrates that cartilage damage is loading-rate-dependent, even when assuming that the damage mechanism is not loading-rate-dependent. At the basis of this phenomenon is the time-dependent behavior of cartilage tissue. Explanations for differences between the damage development in the proteoglycan-rich matrix and the collagen network, as well as peculiar patterns in the collagen damage development, are proposed. These fundamental insights into cartilage damage development are anticipated to be useful for future developments of treatment modalities that prevent progression of cartilage damage.
